# Surgical Cases

**Published:** 1856-12

**Authors:** Daniel Meeker

**Affiliations:** Professor of Anatomy in the Medical Department of the Iowa State University


### IOWA MEDICAL JOURNAL.

VOL. III. KEOKUK, IOWA, NOV. AND DEC., 1856. NO. 6.

### ORIGINAL COMMUNICATIONS.

### SURGICAL CASES.

BY DANIEL MEEKER, M. D.

Professor of Anatomy in the Medical Department of the Iowa State University.

CASE NO. 1.—*Osteo-sarcoma of the Lower Jaw.*—James Casey, of Legro, Indiana, aged 16 years, and of good constitution, had enjoyed good health until he had received a severe blow upon the side of his lower jaw, which resulted in inflammation, swelling and suppuration. The bone soon became involved, some slight exfoliations took place and a discharge of pus of a fœted character continued for sometime. Several small pieces of bone were discharged, after which the external openings healed up. The body and the ramus of the jaw continued to enlarge so as to present a large tumor upon the side of the face. During the progress of the disease, two of the molar teeth became loose and fell out, leaving a rough, irregular opening in the alveolar process, with everted edges, discharging from the cancellous structure of the bone a dark fœted pus, in considerable quantity. His general health, at this time, became involved to such a degree, that he consulted me in the case. I considered it *osteo-sarcoma*, and advised the removal of the jaw of the diseased side, to which the patient consented. The necessary preparations having been made, and the patient having been put under the influence of chloroform, I made an incision along the body of the jaw to the angle, thence along the ramus as high as the articulation, corresponding with the inferior margin of the bone, and then made a vertical incision to the side of the symphysis. The outer surface of the bone was laid bare, the buccinator muscle and mucous membrane of the mouth [divided. The facial artery having been divided was secured by ligature, and the bone entirely separated from the soft parts near the chin, a smooth piece of tin was slipped under it so as to protect the soft parts, and then sawed through with a metacarpal saw. The bone was then seized by a strong pair of forceps, depressed and held by an assistant, the masseter and pterygoid muscles were separated from the bone, and the condyle disarticulated and the bone removed. Cold water was then applied to the wound by sponges for one hour, until all the bleeding had subsided; the parts were then brought together by the pins and the hare-lip suture, the cheek being kept out by lint stuffed in the mouth and internal part of the wound so as to encourage the granulating process. The wound healed kindly and the patient was discharged, cured, in three weeks, with scarcely any perceptible deformity, except the scar along the lower edge of the jaw. The attachment of the digastricus muscle, genio-hyoideus and genio-hyoglossus were preserved, which kept the remaining part of the maxillary from being drawn to one side.

The operation was performed several years since, and the patient still remains in good health, with a perfect use of the jaw, and little or no deformity existing.

CASE 2.—Daniel Baker, aged 62 years, living on Wild-cat Prairie, Tippecanoe Co., Indiana, had had a disease of the shoulder joint of right side, which had been considered chronic rheumatism, until about two years ago, when the joint became swollen and painful. Suppuration ensued and several openings were made, and pus was discharged freely from the joint, making a severe drain upon his system. I was called to see him, and found him at this time very much reduced, and confined to his room, and most of the time in bed. The shoulder was very much swollen, and the arm hung useless by his side. I advised resection of the head of the bone, believing it to be in a diseased condition. He was suffering from night sweats, and loss of appetite, for which I gave him the following constitutional treatment: Sulph quinine and elixir vitriol, followed by comp. ext. sarsaparilla, and iodide potassium, which was continued until some considerable improvement was manifest in his general health. I proceeded to perform the operation as follows: A flap from the deltoid muscle was raised by transfixing it in front of the joint, and raising it so as to expose the capsular ligament, which was divided. An assistant approximated the arm to the body and a small thin piece of wood was insinuated between the soft part and bone, and the upper part of the bone removed by the saw. It was found in a diseased condition at the point sawed through, and I then separated the muscles from the bone lower down, and finding the bone still diseased, I proceeded to remove the arm at the joint. The capsular ligament was very much thickened and diseased; the head of the humerus rough and carious, the glenoid cavity of the scapula also rough and carious and it was consequently cut away with Liston's bone forceps. The axillary artery was now tied, with several smaller branches, which had been controlled by compressing the subclavian above the clavicle during the operation by an assistant. The flap which had been made by the deltoid was now brought down, and the parts brought together by sutures and adhesive straps. The patient was under the influence of chloroform during the operation and suffered but little pain. The wound healed very readily, and nothing unusual occurring during the progress of the case. His health was soon restored and he has since removed to Illinois.

CASE 3.—Otha F. Stingley, of White Co., Indiana, aged twenty-six years, of tall stature and fair skin, light complexion and fine muscular development, was afflicted with a tumor occupying the anterior and superior surgical region of the neck, which developed in size, without much pain, until it extended laterally, so as to press upon the cervical plexus of nerves, and extended downward until it reached the clavicle. His general health now became involved, so that in May following he was compelled to confine himself to his room and soon to his bed. I was called to see him on the 20th of June, and found a large tumor extending from the ear, which was pushed up by it, and passing forward below the lower jaw and resting upon the side of the trachela, to the clavicle posteriorly to the anterior edge of the trapezius muscle, thus occupying nearly all of the surgical regions of the left side of the neck. The tumor felt hard and knotty yet somewhat movable, and was firmly bound down by the sterno-cleido-mastoid muscle, which was spread out and thinned over the surface of the tumor. The rapid development and enlarged condition of the veins upon the surface and side of the neck, with their peculiar livid hue, and sallow countenance which he exhibited, was conclusive evidence, to my mind, that the tumor was of a malignant character. At this time the pressure upon the cervicle plexus of nerves gave him the most exquisite suffering, the pain extending around to the back part of the head, and he became very anxious to have the tumor removed to relieve his suffering. He had, also, a small tumor of the same character, upon the sternum. An operation having been determined upon, the patient was put under the influence of chloroform, and the small tumor over the sternum, removed first. Then an incision was made from the angle of the jaw to the clavicle, thence above the clavicle towards the shoulder and from the angle of the jaw to the ear. After dividing the integument, superficial facia and platisma muscle, I found the sterno-cleido mastoid spread out upon the tumor which was loosened from the surface of the tumor and pushed to one side, and the dissection continued laying bare the sheath of the common carotid artery for nearly its whole length. I then loosened it from below and raised the tumor upwards, dissecting it from the tissues beneath until I arrived at the lower edge of the parotid gland, which I found firmly attached to the tumor, and enlarged and hard. I now proceeded to loosen the gland from the cartilaginous tube of the ear, and then with my finger and handle of scalpel, succeeded in completely removing it. That portion of the gland dipping behind the styloid process was loosened with my finger, and the whole gland removed attached to the tumor. The patient lost considerable blood duing the operation. The principal vessels requiring the ligature, were the facial artery and the external carotid, where it enters the parotid gland; the operation was slow and tedious. The usual dressings were applied. The patient seemed comfortable after the operation, and the wound healed rapidly. He being some eighty miles from my residence, was attended by a physician of the neighborhood, from whom I received the following account:—About two weeks after the wound had healed entirely up, the tumor commenced growing again, and a severe pain in his back, and right hip, also, commenced about the same time, ending in paralysis of the lower extremities. At this time a large tumor could be felt through the parietes of the abdomen, filling up the space between the pubis and umbilicus seeming prominent and hard without any feeling of fluctuation. After the tumor made its appearance, the bowels became very inactive from its pressure. This condition continued until a short time before he died, when the tumor disappeared suddenly and the bowels moved freely. The operation was performed on the 20th of June, and he died the latter part of August following. No *post-mortem* was had in the case so as to develop the true nature of the tumor in the bowels, but it may be presumed that it was a malignat tumor like the one upon the neck, which was of an encephaloid character. This form of malignant tumor is not unfrequently found developed from the different structures within the abdominal cavity.

